# Automatic Detection and Classification of Pole-Like Objects for Urban Cartography Using Mobile Laser Scanning Data

**DOI:** 10.3390/s17071465

**Published:** 2017-06-22

**Authors:** Celestino Ordóñez, Carlos Cabo, Enoc Sanz-Ablanedo

**Affiliations:** 1 Departmento de Explotación de Minas, Grupo de Investigación en Geomática y Computación Gráfica (GEOGRAPH), Universidad de Oviedo, 33004 Oviedo, Spain; carloscabo.uniovi@gmail.com; 2 Grupo de Investigación en Geomática e Ingeniería Cartográfica (GEOINCA), Universidad de León, Avenida de Astorga, s/n, 24001 Ponferrada, Spain; enocsanz@unileon.es

**Keywords:** Mobile Laser Scanner (MLS), point cloud, pole-like objects, automatic feature detection, principal component analysis

## Abstract

Mobile laser scanning (MLS) is a modern and powerful technology capable of obtaining massive point clouds of objects in a short period of time. Although this technology is nowadays being widely applied in urban cartography and 3D city modelling, it has some drawbacks that need to be avoided in order to strengthen it. One of the most important shortcomings of MLS data is concerned with the fact that it provides an unstructured dataset whose processing is very time-consuming. Consequently, there is a growing interest in developing algorithms for the automatic extraction of useful information from MLS point clouds. This work is focused on establishing a methodology and developing an algorithm to detect pole-like objects and classify them into several categories using MLS datasets. The developed procedure starts with the discretization of the point cloud by means of a voxelization, in order to simplify and reduce the processing time in the segmentation process. In turn, a heuristic segmentation algorithm was developed to detect pole-like objects in the MLS point cloud. Finally, two supervised classification algorithms, linear discriminant analysis and support vector machines, were used to distinguish between the different types of poles in the point cloud. The predictors are the principal component eigenvalues obtained from the Cartesian coordinates of the laser points, the range of the Z coordinate, and some shape-related indexes. The performance of the method was tested in an urban area with 123 poles of different categories. Very encouraging results were obtained, since the accuracy rate was over 90%.

## 1. Introduction

Today, efficient and rational management of any sustainable urban environment requires the use of an increasing amount of geospatial information. Mobile laser scanning (MLS) is a relatively new technology that has a promising future as a tool to obtain accurate cartography and construct 3D models, mainly in urban and road environments [1]. The MLS system normally integrates two laser scanners, four to six CCD (charge-coupled device) cameras, one or two GNSS (global navigation satellite system) receivers, an inertial navigation system (INS), a distance measuring indicator (DMI), and software and hardware to collect and process all the data [2]. All these components are placed in a vehicle, so the data are collected while the vehicle is travelling. The main product is a georeferenced 3D point cloud representing the space around the vehicle.

Despite its great capacity to collect data in a short period of time, the fact is that MLS still has some drawbacks that must be gradually corrected to consolidate this technology among cartographers. One of these drawbacks is the cost of the equipment, which is expected to decrease over time. Another weakness of MLS is concerned with the fact that the raw product is a massive heterogeneous and unstructured cloud point which must be processed to obtain useful information for cartographers. As this process is time-consuming, there is a great interest in developing software devoted to generate cartography automatically. Given that MLS is a developing technology, there is still not much literature concerning the development of algorithms to process MLS point clouds, although algorithms developed for terrestrial laser scanner (TLS) data could also be applied. An overview of different techniques for the extraction of surfaces from point clouds, such as scan line segmentation, surface growing, or clustering methods for the recognition of parameterized surfaces, can be seen in [3,4] proposed a sequential method to classify point clouds in real time by means of a hidden Markov model, although they limit their study to distinguish between vegetation and non-vegetation regions, and between vertical and horizontal surfaces in non-vegetation regions. [5] used a link-chain segmentation algorithm and a classification method to establish different classes of objects in urban environments while they assume that the ground is flat. The classification is based on comparing previously segmented objects with a set of standard, predefined thresholds. Other interesting works related to mobile laser scanning segmentation in urban or road environments, not specifically concerned with pole-like objects, can be seen in [5,6,7,8,9,10,11,12]. The segmentation and classification of urban features can also be performed using data from image-based devices. Segmentation can be implemented directly from the images (e.g., [13,14]), or using dense point clouds derived from them (e.g., [15,16,17]).

Pole-like objects, such as lampposts, traffic lights, and street trees, are urban elements of interest for cartographers, and consequently some algorithms have been developed for the automatic or semi-automatic detection and/or classification of these objects using MLS point clouds. [18] presented a method for organizing objects (cars, traffic lights, fire hydrants, lampposts, etc.) in 3D point clouds of urban environments obtained by combining MLS and ALS (aerial laser scanning) systems. Once the objects have been detected throughout segmentation, they are classified in different categories using different machine learning techniques such as k-nearest neighbors and support vector machines. The recognition rates are not very high and, moreover, at this moment, the interest is principally in unsupervised methods that do not require operator intervention. [19] detected vertical pole-like objects in a road environment using MLS point clouds by means of a sequential algorithm that includes scan line segmentation, clustering, and classification. The average correctness of the detection was 81.0%. [20] employed a covariance-based procedure to perform a segmentation of MLS point clouds to detect road poles. [21] recognized pole-like objects from MLS point clouds using a Laplacian smoothing followed by principal component analysis for recognizing points on pole-like objects. Pole-like objects with various radii and tilt angles were recognized. [22] developed a method to detect basic structures (trees, traffic signals, lampposts) from MLS data that uses several parameters such as size, shape, orientation, and topological relationships. [23] used voxelization and morphological analysis to detect street trees and some morphological parameters from MLS data. [24] presented an algorithm to detect and classify traffic signs from MLS data. The classification is based on 2D images using a Gaussian-Bernoulli deep Boltzmann machine-based hierarchical classifier. [25] developed a method for the detection and recognition of retro-reflective vertical traffic lights from MLS data. First, the traffic lights are extracted from the point cloud according to geometric and radiometric information. Then, the resulting cluster of points is rasterized and classified in different categories using a linear regression. 

In this paper, we develop an algorithm for the automatic detection and classification of five types of pole-like objects from MLS point clouds based on the previous work of [26]. The authors proposed a methodology to detect pole-like street furniture objects based on discretizing the cloud point in voxels and analyzing horizontal sections of the voxel, but although they obtained good results, they did not tackle the problem of classifying the pole-like objects into categories. The distinction between the said elements is carried out using a discriminant analysis algorithm that groups the objects based on the principal component scores obtained from the Cartesian coordinates of the points and on three indexes that are related to the shape of the objects.

## 2. Materials and Methods

### 2.1. Data Acquisition

The methodology developed in this paper has been tested on a set of data collected with the Lynx Mobile Mapper system (Teledyne OPtech, Toronto, ON, Canada). The system is based on two LIDAR (light detection and ranging) sensors mounted on a vehicle, an inertial measurement unit (POS LV 520) produced by Applanix (Markham, ON, Canada), which consists of a two-antenna heading measurement system, and an inertial navigation system.

The LIDAR sensors are located in the rear part of the van. Each of them registers points in a plane at 60° to the horizontal and 45° to the longitudinal axis of the vehicle (i.e., the driving direction) with a 360° field of view. The scan frequency of these sensors varies from 80 to 200 Hz, and the pulse repetition rate (PRR) ranges from 75 to 500 kHz. The speed of the van was approximately 40 km per hour. According to the manufacturer, the maximum range is 200 m (20% reflectivity), and the absolute precision of the points registered is ±5 cm (1σ).

**Figure 1:**
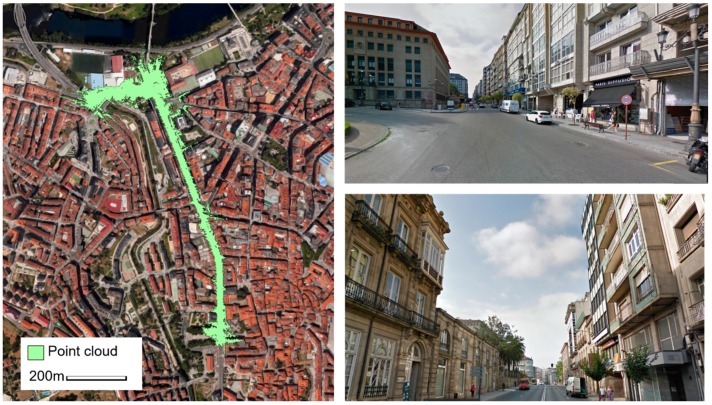
Plan view of the point cloud (**left**) and two photographs of the street where the point cloud was collected (**right**).

The MLS point cloud was collected in a street in Ourense, a city in the northwest of Spain (Figure 1). It contains 203 million points along 1 km (i.e., on average, 200,000 points per meter along the trajectory of the MLS). The street contains different pole-like objects, particularly trees, lampposts, traffic lights, traffic signals, and traffic panels. The aim of this paper was to detect these objects and separate them into categories. 

### 2.2. Point Cloud Segmentation and Classification

#### 2.2.1. Segmentation

Segmentation of the MLS point cloud was first carried out in order to distinguish pole-like objects from other objects such as buildings, vehicles, or the ground. The segmentation algorithm follows a previous work ([26]). It consists of three consecutive steps: (i) voxelization of the point cloud space; (ii) two-dimensional analysis of horizontal sections of the voxelized point cloud; and (iii) tridimensional reconstruction of the selected features from the previous 2D analysis. In cases where the point cloud is too large for computing the whole dataset at once, the point cloud is automatically divided into smaller subsets with spatial overlap. This division can be performed by splitting the extent of the point cloud into overlapping rectangles, or into stripes along the trajectory of the vehicle. The different subsets are analyzed separately, and afterwards, duplicate pole-like objects detected in the overlapped areas are joined or eliminated.

Voxelization consists of discretizing the space in a regular tridimensional grid of cubes, and it is used to reduce the amount of data and the processing time. For each of these cubes (voxels) only the coordinates of its centroid and the number of points inside it are stored, given that only this information is needed in the following stages. Moreover, a one-to-one correspondence between voxels and laser points is established. 

**Figure 2:**
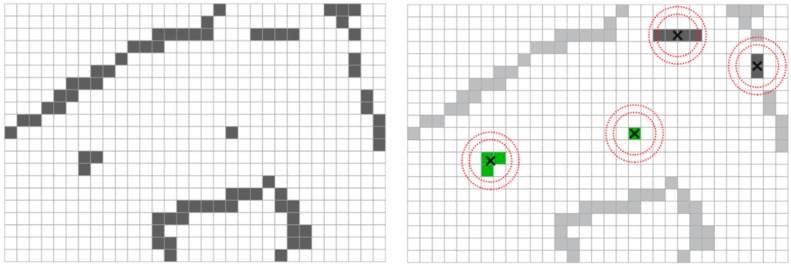
Two-dimensional analysis for pole detection. (**Left**): Horizontal layer of the voxelized space containing poles and other objects; (**Right**): Groups of voxels that fulfill all the requirements (green), small groups that do not overcome the isolation filter (dark grey), and large groups of voxels (light grey).

Once the voxelization has been performed, the tridimensional grid is divided into horizontal slices in order to identify the structures that most likely correspond to the horizontal section of a pole. The identification is carried out by a neighborhood analysis assuming that poles are isolated objects with a small horizontal section. From the centroid of each cell a ring is defined. Poles are searched for by detecting cells inside the ring. The number of points located inside the ring is calculated and a section is rejected when a threshold is surpassed. Figure 2 shows different situations that can take place during the search process. 

**Figure 3:**
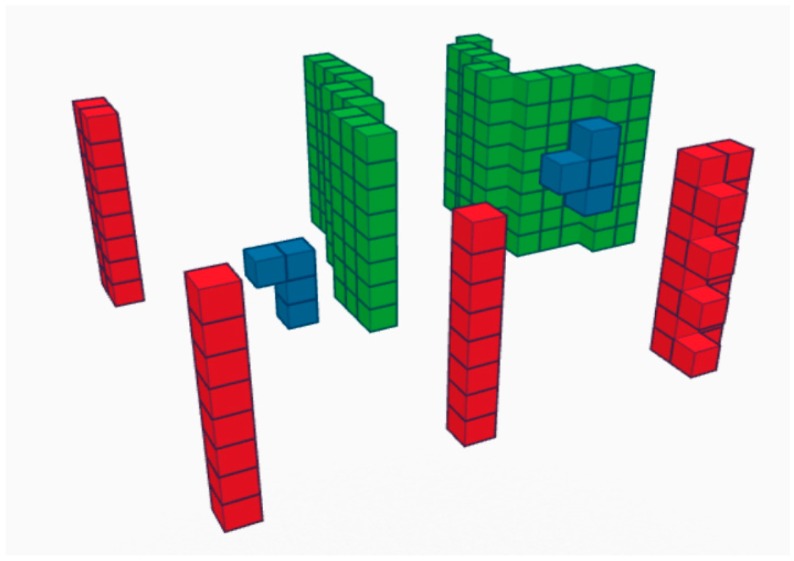
Three-dimensional assembling and analysis. Green groups: voxels in sections that do not fulfill the isolation requirements; Blue groups: voxel groups with valid sections, but without vertical continuity or enough height; Red groups: poles.

The third stage consists of assembling the cell candidates into poles. All the voxels that share a face, an edge, or a corner are grouped together. Furthermore, vertical continuity and a minimum height are required for these voxel structures to be considered as poles (Figure 3).

#### 2.2.2. Classification

##### Methods

**Figure 4:**
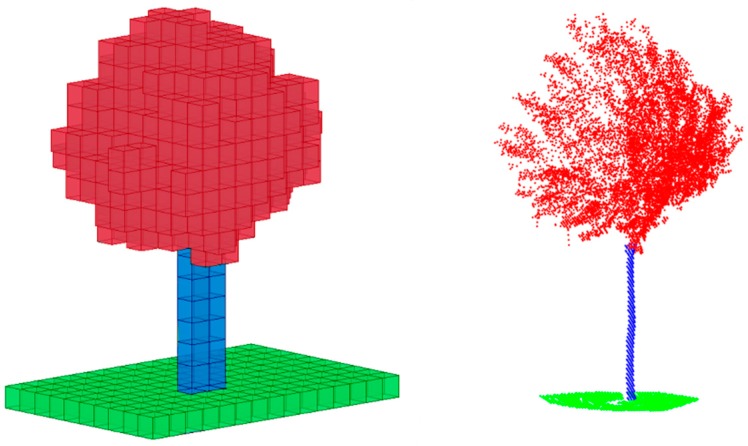
Pole object extraction. (**Left**): group of voxels defining the actual pole (blue), linked voxels conforming the object attached to the pole (red), and voxels containing points on the ground (green); (**Right**): points inside each voxel group that inherit the voxel labels.

Once the poles have been detected, we are interested in classifying them into six categories present in the scene: Trees (1), lampposts (2), advertising signals (3), small traffic lights (4), large traffic lights (5), and traffic signs (6). In order to do this, we first extract each of the objects and then apply a supervised classification algorithm. Each of the sample objects was extracted from the point cloud by first detecting the actual pole, as explained in the previous section, and then detecting the top part. All the voxels connected to the top section of each pole are grouped and labeled. Then, using the reversible nature of the voxelization algorithm [26], the points inside the labeled voxels are extracted (Figure 4). These points inherit the labels from the voxels that contain them, and conform the sample objects.

The classification into different categories was carried out using the well-known linear discriminant analysis (LDA) ([27,28,29]) and support vector machine (SVM) ([30,31,32,33]) techniques for multiple classes, which have been largely applied in many different disciplines. There are many other classification algorithms, but we opted for LDA because of its simplicity, and SVM, for its capacity to determine non-linear boundaries between classes. 

LDA is a linear transformation technique that computes the directions representing the axes that maximize the separation between multiple classes. Given a set of observations $\left(x_{1},x_{2},\ldots ,x_{n}\right);\;x_{i}\;\in \;{\mathbb{R}}^{d}$, *d* being the number of variables, each of them belonging to a class $y_{c},\;c=1,..,C$, the aim of the LDA is to find, for a new observation $x_{l}$, a good predictor $y_{c}$ of the class it belongs to. Assuming that each of the *C* classes has a mean $\mu_{j}$, the within-class and between-class scatter matrices, $S_{w}$ and $S_{b}$, respectively, are

$$S_{w}=\sum_{j=1}^{C}\sum_{i=1}^{N_{j}}\left(x_{ij}-\mu_{j}\right){\left(x_{ij}-\mu_{j}\right)}^{T};\;S_{b}=\frac{1}{C}\sum_{j=1}^{C}\left(\mu_{j}-\mu \right){\left(\mu_{j}-\mu \right)}^{T}$$

where μ is the mean of the class means, $x_{ij}$ is the *i*th sample of class *j*, and $N_{j}$ is the number of samples in class *j*. The goal is to maximize the between-class measure while minimizing the within-class measure. One way to do this is by maximizing the ratio of the scatter matrices determinants $R=\frac{\left|S_{b}\right|}{\left|S_{w}\right|}$.

If $S_{w}$ is a non-singular matrix, this can be accomplished by calculating the eigenvectors of $S_{w}^{-1}S_{b}$ corresponding to the largest eigenvalues. These eigenvectors represent the directions of maximum separation between classes.

The multiclass SVM classifier is an extension of the original binary classifier (y={−1,1}) which looks for the maximum-margin hyperplane, that is, the hyperplane representing the largest separation between the two classes. The algorithm is based on mapping the original finite-dimensional space into a much higher-dimensional space where the boundaries between classes are linear. A hyperplane can be written as the set of observations satisfying wx+b=0, where w represents the normal vector to the hyperplane and *b* represents the offset. Binary SVM tries to find two parallel hyperplanes equidistant from the maximum-margin hyperplane which separates both classes while the distance between them is as large as possible. Analytically, the solution is formulated as the following minimization problem with restrictions:

$$\left\{ \begin{array}{l} \underset{w,b}{\min}\frac{1}{2}{\left\|w\right\|}^{2}\\ y_{i}\left(wx_{i}+b\right)\geq 1,\;i\in \left\{1,...,n\right\} \end{array}\right.$$

The solution is a linear combination of a subset of observations near the margin hyperplanes denominated support vectors. Those observations $x_{i}$ located near the margin hyperplanes completely determine them, and for that reason they are called support vectors (s.v.):

$$w=\sum_{s.v.}\beta_{i}x_{i}$$

In this work, we used a mathematical approach based on kernels to solve the SVM model. In particular, a radial basis function (RBF) kernel was used. Given two samples $\left(x_{i},x_{j}\right)$, the RBF kernel is $K\left(x_{i},x_{j}\right)=\exp\left(-\gamma {\left\|x_{i}-x_{j}\right\|}^{2}\right)$, $\gamma =\frac{1}{2\sigma^{2}}$ being a parameter that measures the radius of influence of samples selected by the model as support vectors. 

Apart from γ, there is another parameter in SVM that needs to be considered in order to obtain an optimal solution, and it is usually referred as *C*. *C* is a generalization parameter that allows the modification of the number of samples selected as support vectors. It controls the tradeoff between smooth decision boundary and classifying the training points correctly. When *C* is too large, errors are highly penalized in the classification, but the model may overfit. On the other hand, a too small *C* value may cause underfitting.

##### Predictor Variables

Except for one variable, the rest of the predictor variables used in the classification are related to the eigenvalues obtained from a principal component analysis (PCA) applied to the coordinates of the laser points. For each of the pole-objects in the sample, the eigenvalues were calculated through an eigendecomposition of the covariance matrix ∑ of the laser points $p_{i}=\left(X_{i},Y_{i},Z_{i}\right)$:

$$\sum =\sum_{i=1}^{N}{\left(p_{i}-\bar{p}\right)}^{T}\left(p_{i}-\bar{p}\right)$$

where *N* is the number of laser points of the object.

After testing several variables related to the geometric characteristics of the objects under study, finally only four $x_{j}\;;j=1,...,4$, those corresponding to the simplest model of high accuracy, were selected (the discarded covariates tested were λ_1_, λ_2_, and λ_3_, the range of λ_1_, λ_2_, and λ_3_, and the volume of the convex hull). They were specifically the following:

$x_{1}=\;\mathrm{range}\;\mathrm{of}\;Z\;\mathrm{coordinate}$. Discriminates between small (traffic signals) and large objects (lampposts, advertising signals).
$x_{2}=\frac{\lambda_{3}}{\lambda_{1}\lambda_{2}}$. Discriminates flat elements (traffic signals) from other objects.
$x_{3}=\frac{\lambda_{2}}{\lambda_{3}}$. Distinguishes narrow objects, such as traffic signals, from wider elements with similar values of $x_{2}$, such as lampposts.
$x_{4}=\frac{\lambda_{1}\lambda_{3}}{\lambda_{2}^{2}}$. This variable discriminates between volumetric objects (trees) and flatter objects (traffic signals or lampposts).

where $\lambda_{i},\;i=1,..,3$, are the three eigenvalues obtained from a PCA (principal component analysis). 

**Figure 5:**
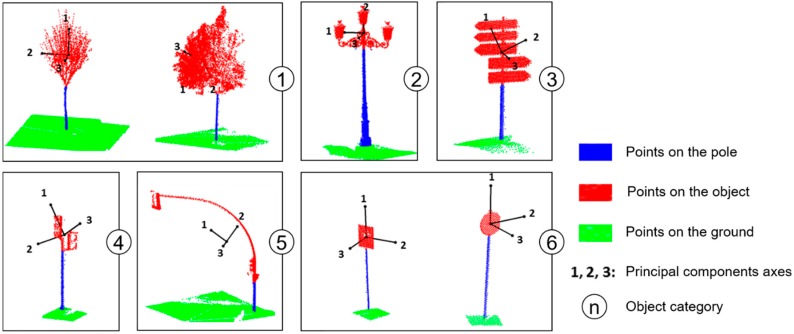
Pole-like object categories extracted from the point cloud.

Figure 5 shows a sample of the different kinds of pole-like objects extracted from the MLS point cloud. The base is blue and the top part is red. The eigenvectors are also represented in order to show their relationship with the geometry of the objects. 

As can be appreciated in Figure 5, the planar traffic signal has two eigenvalues, corresponding to the eigenvector contained in the plane, which are greater than the third one, which corresponds to the eigenvector normal to that plane. Accordingly, we expect to obtain small values of $x_{2}$ for these objects. Lampposts are in some way similar to traffic signs, but they are wider, and then they have a larger value of the variable $x_{3}$. For trees, especially when in leaf, the three eigenvalues tend to have similar values, so we expect to obtain larger values of variable $x_{3}$ than for the rest of the objects. As was mentioned, variable $x_{1}$ was mainly defined to distinguish between small and large objects.

## 3. Results

The segmentation and classification methods previously explained were applied to the point cloud described in Section 2.2. All the methods were implemented in Matlab, and the analysis was performed on a commercial laptop (N-551-JB: Core i7, 2.6 GHz, 8 GB DDR3, Asus, Taipei, Taiwan). The point cloud was automatically divided into four overlapping stripes (i.e., each stripe contains points registered by the sensors from a 265-m segment along the trajectory, and the overlap between consecutive stripes is 20 m). The segmentation algorithm was applied individually to the four stripes using a voxel size of 10 cm, and then duplicated pole-like objects were removed. Processing time for the segmentation was 486 s, and 0.2 s for the classification (once the model parameters were determined). The segmentation algorithm detected 91% of the total number of poles in the point cloud, and only 3% of the objects were detected as poles although they were not. Most of these misdetected poles were almost-vertical branches, so the error could have been avoided by increasing the outer diameter in the segmentation process. As a result, we obtained a sample data with 153 pole-like objects. As this was an unbalanced sample, since there were a greater proportion of trees than other types of objects, we finally removed 30 trees. Accordingly, we obtained a sample of 123 objects with almost the same proportion of elements of each category.

Table 1 shows the confusion matrix obtained after applying LDA and SVM to a sample test formed by 40% (49 objects) of the sample data (the remaining 74 objects were used to train the algorithms). In order to select the optimal values for parameters γ and *C* of the SVM classifier, a cross-validation procedure was applied to the training dataset for each pair of values $\left(\gamma_{j},C_{j}\right),j=1,...,m$ on a grid in the interval [2^−10^, 2^10^]. Columns refer to existing pole-types and rows refer to predicted pole-types. The last column represents errors of commission (EC, or the ratios of poles classified as a type that is different from the true type) and the last row represents errors of omission (EO, or the ratio of poles representing a specific type that have been classified in another category). The last two rows register the overall accuracy (ACC) and the kappa index (*k*). ACC is the fraction of correctly classified poles with regard to all the poles in the sample. *k* is also a measure of the accuracy in the classification, but it takes into account the possibility of the agreement occurring by chance:

$$k=\frac{\mathrm{observed}\;\mathrm{accuracy}\;-\;\mathrm{change}\;\mathrm{agreement}}{1\;-\;\mathrm{chance}\;\mathrm{agreement}}$$

Thus, the results obtained confirm that the four variable predictors used to classify the objects are appropriate, given the high accuracy reached with both classification models.

After repeating the analysis with different training-test samples, it was found that class 3 (advertising panels) was, in general, the worst classified. In fact, we detect a trend whereby some advertising panels are misclassified as lampposts or traffic signs.

**Figure 6:**
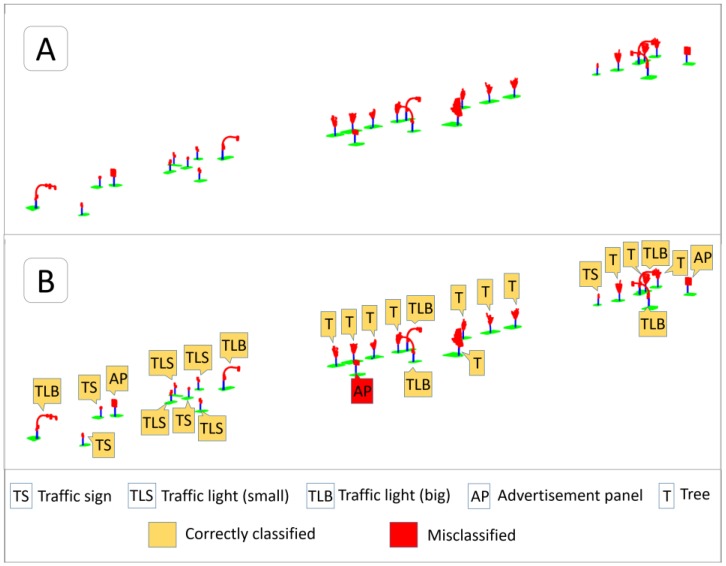
Pole-like object segmentation and classification in a stretch of the test street. (**A**) Poles, features attached to them, and a portion of ground around each pole following the same color schema as in Figure 5; (**B**) Classification and labeling of the objects on segmented poles using SVM.

Figure 6A shows the distribution of 28 poles and the objects attached to them in a section of the test street. Figure 6B presents the results of the SVM classification on these objects, including an advertisement panel classified as a traffic sign. This misclassification could be due to the reduced size of this specific panel, whose size and shape are similar to some rectangular traffic signs. 

## 4. Conclusions

A methodology for the recognition of different types of pole-like objects from mobile laser scanning point clouds was developed. First, we detect the objects using a heuristic segmentation algorithm that assumes that poles are isolated and almost vertical objects. Secondly, linear discriminant analysis (LDA) and support vector machine (SVM) algorithms were used to distinguish between six types of pole-like objects. 

After trying several shape-related predictor variables to carry out the classification of the objects, we finally select only four of them, consisting of those that provide the best prediction with the minimum number of variables. One of those variables is the range of the *Z* coordinate and the other three are simple algebraic expressions that relate the eigenvalues of a principal component analysis (PCA) conducted on the Cartesian coordinates.

The application of our methodology to a dataset produced very good results for both models (LDA and SVM) with very low error rates and high kappa indices. Errors of commission and omission were also very low, with the class of advertising panels providing the worst results.

## Figures and Tables

**Table 1:** Confusion matrix for the LDA and SVM models.

LDA								SVM							
	1	2	3	4	5	6	EC		1	2	3	4	5	6	EC
1	10	0	0	1	1	0	0.17	1	11	0	0	1	0	0	0.08
2	0	8	0	0	0	0	0.00	2	0	8	0	0	0	0	0.00
3	0	0	3	0	0	0	0.00	3	0	0	3	0	0	0	0.00
4	0	0	0	10	0	0	0.00	4	0	0	0	10	0	0	0.00
5	0	0	1	0	4	0	0.20	5	0	0	0	0	5	0	0.00
6	0	0	0	0	0	11	0.00	6	0	0	1	0	0	10	0.09
EO	0.00	0.00	0.25	0.09	0.20	0.00	0.06	EO	0.00	0.00	0.25	0.09	0.00	0.00	0.04
ACC	0.94							ACC	0.96						
*k*	0.93							*k*	0.95						
